# Estimating a Path through a Map of Decision Making

**DOI:** 10.1371/journal.pone.0111022

**Published:** 2014-11-04

**Authors:** William A. Brock, R. Alexander Bentley, Michael J. O'Brien, Camilia C. S. Caiado

**Affiliations:** 1 Department of Economics, University of Wisconsin, Madison, WI, United States of America and Department of Economics, University of Missouri, Columbia, MO, United States of America; 2 Department of Archaeology & Anthropology, University of Bristol, Bristol, United Kingdom; 3 Department of Anthropology, University of Missouri, Columbia, MO, United States of America; 4 Department of Mathematical Sciences, Durham University, Durham, United Kingdom; US Army Engineer Research and Development Center, United States of America

## Abstract

Studies of the evolution of collective behavior consider the payoffs of individual versus social learning. We have previously proposed that the relative magnitude of social versus individual learning could be compared against the transparency of payoff, also known as the “transparency” of the decision, through a heuristic, two-dimensional map. Moving from west to east, the estimated strength of social influence increases. As the decision maker proceeds from south to north, transparency of choice increases, and it becomes easier to identify the best choice itself and/or the best social role model from whom to learn (depending on position on east–west axis). Here we show how to parameterize the functions that underlie the map, how to estimate these functions, and thus how to describe estimated paths through the map. We develop estimation methods on artificial data sets and discuss real-world applications such as modeling changes in health decisions.

## Introduction

In studies of decision-making and health, social influence is becoming increasingly recognized. Coordinated behavior has benefits for groups and the individuals within them. When successful behaviors of the community are socially learned, cooperation can evolve in social networks extending beyond the limits of Hamiltonian inclusive fitness among kin [1]–[8]. Provided that some fraction of agents learn individually [9], either as “specialists” or “generalists” [10], social learning can be seen as an adaptive strategy among “scroungers” for the exploitation of the information gains made by the “producers” who track the environment through individual learning [11]–[13]. Most evolutionary approaches expect the most adaptive state to equilibrate to a mix of individual and social learners whose proportions are dictated by the degree of spatial and temporal autocorrelation of the environment and the cost of individual learning [7], [12], [14]–[18]. This assumption of adaptive equilibrium is an ideal, however, and not necessarily attainable in conditions of continual transition. As social learners increase in frequency, they are increasingly copying from each other, and so the quality of their information about decision payoffs likely diminishes [12]. At the same time, individual learners may be overwhelmed by rapid change, poor information, or simply too much information in order to make informed decisions.

For this reason, there is the important factor of how well informed decision makers are — what we might call the “transparency” of payoffs in their decisions. A relevant question about online social media, for example, is whether their searchability makes decision makers more well informed, or whether the deluge of social influence and similar options makes decisions less transparent in terms of payoffs [19]. Traditional decision theory typically assumes that agents are informed about their behavioral options, or if not, then are at least knowledgeable about the people from whom they might learn, preferably the most skilful, informed, or prestigious members of the group [12], [20]–[22]. In contrast, models of collective flocking or herding behavior assume no such knowledge — agents are often represented by vectors, with choice as the direction and transparency as the magnitude. Even as most agents follow neighbors with no particular preference, a collective direction (consensus) can nonetheless favor of the minority, if there exists high transparency of choice [23]–[25].

**Figure 1:**
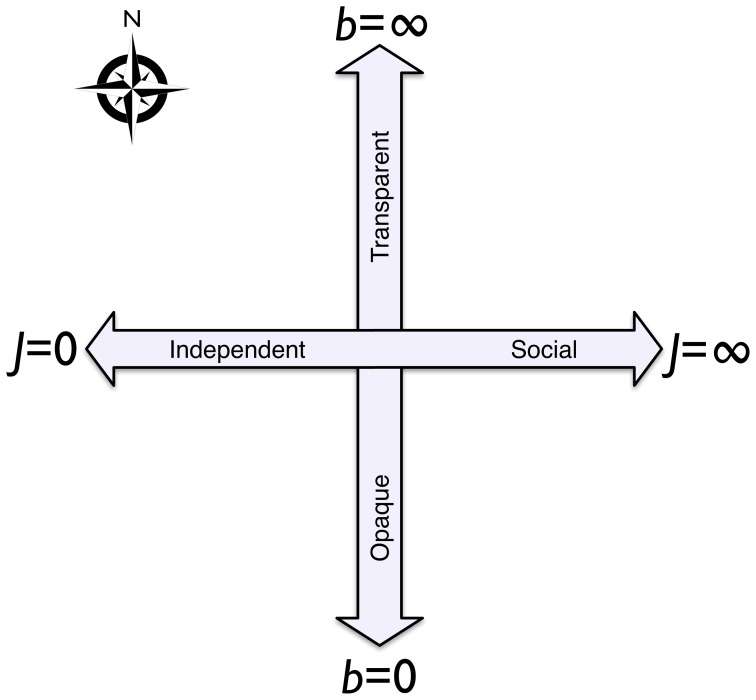
Summary of the four-quadrant map for understanding different domains of human decision making, based on whether a decision is made independently or socially and the transparency of options and payoffs [19].

We see two major factors in decision making: social/individual learning and transparency of choice [19], as depicted in Figure 1. This heuristic map represents the relative magnitude of social versus individual learning on the horizontal axis and the transparency of a decision on the vertical axis. Following the call for evolutionary theory as the integrating principle of behavioral science [26], the map is intended to unify quantitative approaches from multiple branches of social science, ranging from rational-actor approaches in the northwest, to more anthropological social-learning theory in the northeast, to the “information overload” of the southwest and southeast.

At the macro scale, the map reduces the complexity of social decision process analysis to the coarse-grained simplicity of two axes, analogous to a principal components analysis reduced to two dominant factors. The north–south axis, which we parameterize as 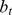, represents a measure of transparency in the payoff differences among available alternatives, from opaque at the south (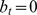) to absolutely transparent in the north (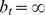). Along the east–west axis, the measured parameter 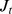 increases from west to east, from a decision made individually at the western edge (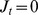) to pure social decision making — copying, for example — at the eastern edge (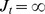).

The framework has broad applicability, and one particular application we envisage is toward fertility decisions for example, using an exceptional long-term dataset on about 250,000 people, collected in the Matlab region of Bangladesh since 1966 [27]. Those data are excellent, long-term monthly records of the decisions that have been made over many years, along with associated (anonymous) details of the individuals making those decisions, such as total fertility, religion, surviving children, age at marriage, household income, education and other observable covariates that impact fertility. We can also consider social variables as well, such as density of the behaviour within the local social network. Other health-related examples would be smoking, where national health services such as the NHS in the United Kingdom or the ALSPAC dataset at University of Bristol hold long-term data on anonymous individuals and their relevant binary choices (to smoke or not, be vaccinated or not), along with a wealth of covariate information on the individuals (wealth, education, religion, and so on) but also often on social visibility (e.g., kin members in the same dataset).

Work on peer effects in smoking behavior is vast, but we have not found any work that attempts to estimate the dynamics of the intensity of choice function, as we propose. Work that is most closely related to ours [28], [29] attempts to control for the effects of self-selection into peer groups, correlated unobservables, and contextual effects that tend to bias received estimates of peer effects on smoking (e.g., estimates of our 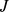 parameter). This valuable precedent, however, does not estimate the intensity of selection function as we propose.

Our approach could be applied to far different scenarios than health, including criminal records or consumer sales, where long-term choice data are available alongside individual covariates. We see the framework as especially appropriate to online choices in the big-data era, as the covariate data could be comprehensive, including vast records of previous choices. In all cases, the characterization on social influence *and* transparency of choice would provide a novel insight into the decision dynamics at the population scale.

We previously described the map in terms of generalized data patterns diagnostic of each of its four quadrants [19]. We focused on population-scale data patterns and left specific empirical estimation concerning individuals to future work. Here we show how real-world data could be plotted as locations on the map in Figure 1 and, if the data allow, as trajectories across the map through time. This requires us to develop a method to estimate 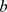 and 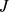, either for each agent or for each agent's group, from real-world data. We assume the available data include the (a) covariates that may influence the agent's choice, (b) variability of the agent's choices, and (c) strength of social influences upon the agent's choices. All three of these associations may change through time.

## The Model

In parametarizing our two-dimensional map, we divide transparency of choice into separable components for intrinsic utility and social influence. Our model builds on previous work in discrete choice theory by parameterizing the transparency of choice as a function of observable covariates (see Methods). To begin, let there be 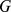 groups with 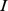 players in each group. We can think of 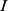 as being a large number so that the law of large numbers gives a good approximation in what follows. For now, assume the groups are disjoint, i.e., nonoverlapping. Agent 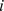 in group 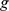 chooses choice 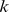 if the random utility agent 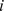 gets from choice 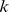 is greater than the random utility available from any other choice,
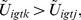
(1)at time 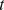 for all 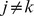. Here, symbols with a 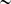 denote random variables (deterministic quantities will not have tildes).

Assume that
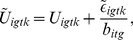
(2)where the 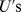 are deterministic and the random variables 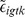 are Independent and Identically Distributed Extreme Values (IIDEV) across all dates, choices, groups, and individuals. Then the transparency of choice is inversely proportional to how strongly the noise in the payoff is amplified, 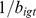. We then choose units so the constant of proportionality is one (so that when noise is small, transparency is high). As we will see, this noise can occur in intrinsic utility and/or social utility of the choice. The probability, 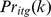, that agent 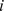 in group 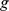 chooses 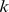 is then the term for choice 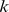 divided by the sum of terms across all choices, 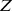:
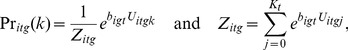
(3)where 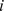, 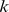 and 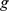 take the integer index values from 1 to 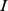, 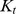, and 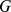, respectively. The higher the transparency of choice is, the more sensitive the probability is to the utility. Note that when transparency of choice 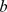 is zero, utility has no effect on choice, and agents are effectively just guessing among all the choices, i.e., 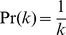 when 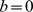.

In order to incorporate the “east-west” axis of social influence, one option is to add a term for frequency-dependent social learning [30], whereas another is to add a social component by which agent 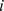 makes pairwise expectations on choices of 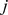 others [31]. Building on both, we define utility with respect to choice 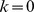 and then divide 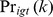 by 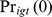, so that the partition function, 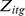, cancels out from equation 3, such that
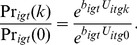
(4)If we then take the natural logarithm of both sides, we are left with the transparency 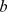 multiplied by the difference in utility 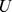. We can then expand the utility function into an individual component and a social component as follows:
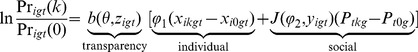
(5)for agent 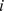, for choice 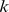, in group 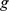, at date 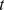 (recall that 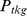 is the fraction of group 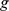 that chose 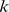 at date 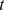).

Table 1 in the Methods section (below) summarizes the different parameters and variables involved. Equation 5 separates, from left to right on its right-hand side, an individual-choice component, 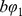, and a social component, 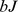. The individual component of choice is governed by 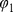 and acts on the payoff difference between options 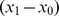. The social-influence component, governed by 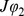, acts on the popularity of the option 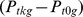 that is expressed as the relative popularity of choice 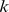 compared to the choice of reference 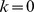. Although the transparency of choice parameter, 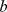, is part of both the individual term and the social-learning term, our map depicts the transparency of choice and social influence as orthogonal dimensions.

**Table 1:** Parameters and variables of the model.

Parameter or variable	Description
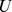	Total utility payoff function (total)
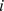	Individual index, from 1 to 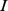
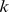	Choice index, from 1 to 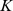
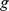	Group index, from 1 to 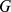
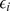	Random idiosyncrasies (noise) associated with agent 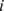
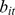	transparency of choice for agent 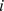 at time 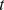 . Depends on 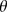 and 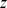 .
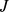	Social influence for agent 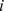 at time 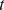 . Depends on 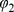 and 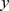 .
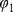	Individual sensitivity to differences in choice 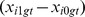
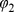	Social choice transparency (parameter vector)
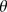	Covariates for choice transparency (parameter vector)
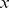	Scalar for individual choice
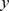	Scalar for presence of social influence
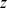	Scalar for variability of choices through time
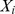	Observable characteristics of agent 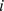 , consisting of 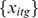

The model is intended to allow the estimation 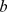 and 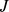 from the data and potentially map a trajectory through time for agent 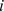 in group 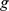. The transparency of choice, 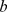, increases from south to north on the map and the social influence, 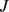, increases from west to east (Figure 1). We may estimate 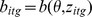 once we have adequate time series data set on a vector of covariates, 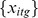, and we have parameterized the transparency of choice function, 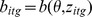. The parameter vectors 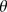 and 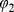 can be normalized to fit the functional specification, 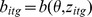, and social-utility function, 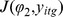, respectively (discussed below).

The covariates for each agent include those that predict the propensity of the behavior, denoted by 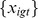, those associated with the presence of social influence 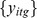, and those that relate how variable the choices were through time, 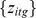. These realities are amplified by 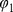 (individual) and 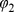 (social), which govern the sensitivity to inherent differences of the choice and social influence, respectively. In other words, the parameter vectors 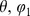, and 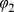 operate on aspects of the real world denoted by positive scalars 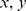, and 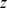, respectively. Estimating the parameter vector 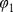 determines the individual sensitivity to differences in choice 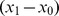. Estimating the parameter vector 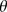, along with the scalar observable 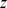, determines the transparency of choice, 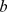. Estimating the parameter vector 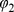 specifies the social-influence function, 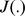.

We tested to see how these estimates can be used to describe a path, 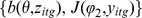, through the map for each agent 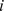 in each group 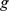 for which we have data at date 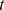.

## Results

**Figure 2:**
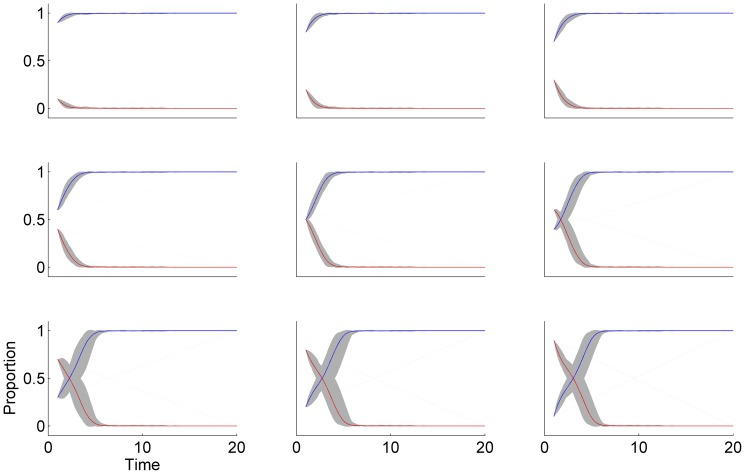
Simulations of 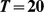 **time steps, with group size** 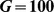 **and** 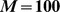 agents per group. From equation 7, we set 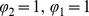, and 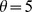. The noise component, 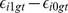 has mean 0 and 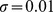. Shown are proportions of the 10,000 total agents who have made one of the two possible two choices, one shown as red and one shown as blue, through time. The different plots show simulations with varying starting points for each proportion. The payoffs 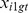 and 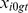 are chosen from from time-varying normal distributions 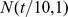, and 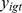 and 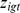 are both chosen from time-varying normal distributions 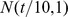. Gray bounds show 95% quantiles for sample paths over group and red/blue curves show the mean paths for proportion over groups.

We generated artificial data to yield four different paths through the map to test whether our suggested estimation procedure actually works (see Methods). We can use equation 5 for a log odds regression,
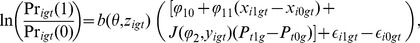
(6)where 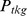 is the fraction of group 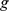 that chose 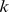 at date 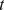. We then specified the social-influence and transparency of choice functions as follows, 
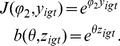
(7)We can now use this parameterisation to explore how the parameterised map applies to artificial datasets (see Methods on how these data were generated). In Figure 2, we show simulations of the binary choice (e.g., to have a child or not) with 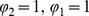, and 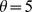 from equation 7, with group size 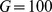 and 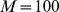 agents per group. We then vary the initial starting proportions of the 10,000 agents (over all groups) choosing one (blue) versus the other (red). We can see that all simulations converge to nearly 100% of agents choosing the blue option in fewer than ten time steps, even if we start with a majority choosing red at the starting point. This is what we expect when both 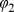 is positive and 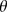 is high and positive — the population selects the option with the better payoff.

**Figure 3:**
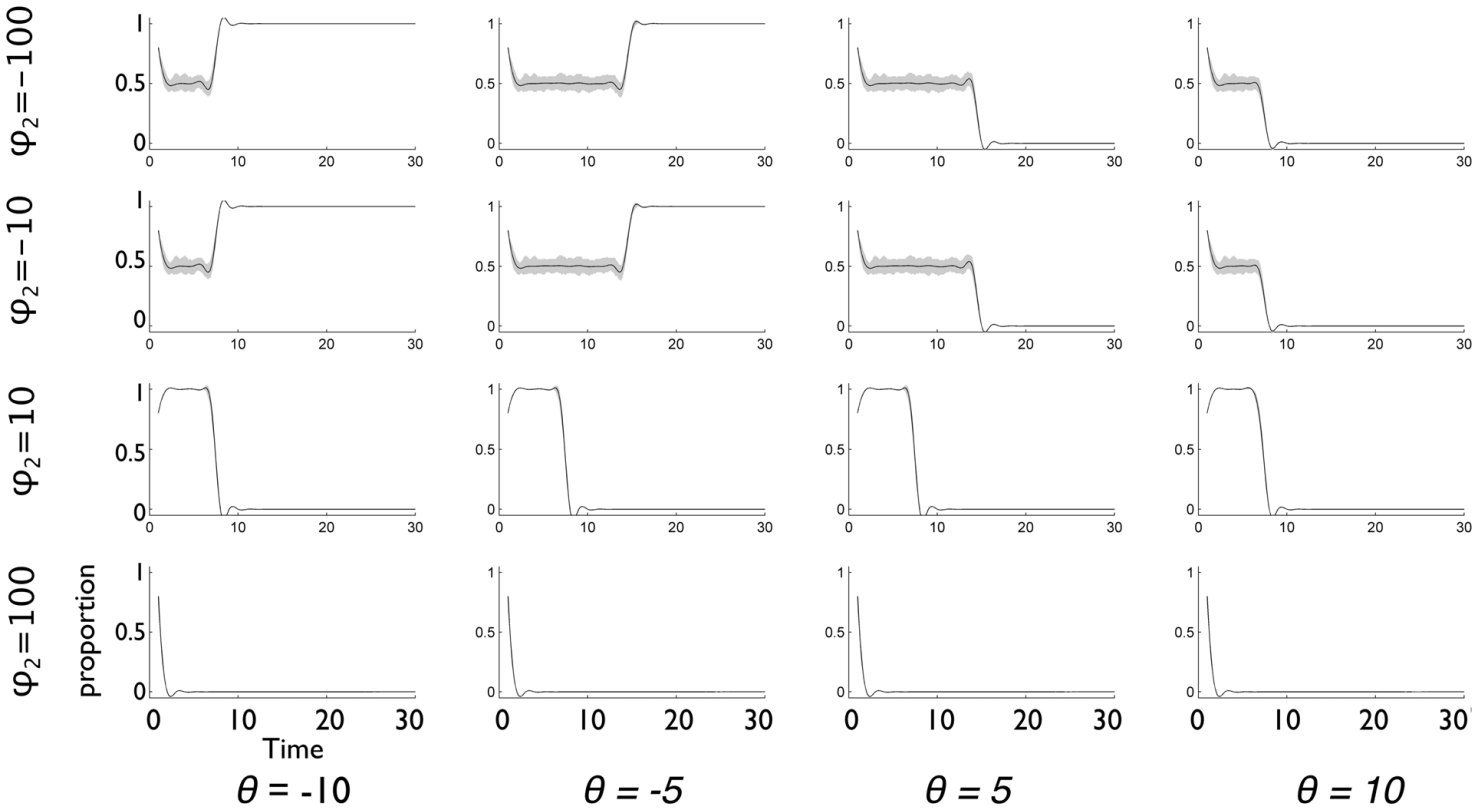
Simulations of binary choice model with varying choice intensity 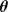 and social influence intensity 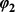. For clarity the plots only show the proportion of agents making one of the two choices (e.g., non-parent). The panels show 16 different combinations of 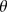 and 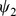, with 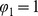 for all. Each panel shows results of simulation with 30 time steps, 100 groups and 200 agents per group, noise component 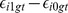 with mean 0 and 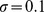, and starting proportion 80% for the choice shown (so the choice not shown starts at 20%). The payoffs 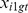 and 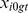 are chosen from 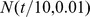, and 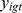 and 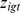 are both chosen from 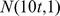. Gray bounds show 95% quantiles for sample paths over group for the proportion of non-parents over groups.

The specification in equation 7 allows other variations that yield more novel results. To convey the effects of varying 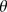 and 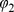, Figure 3 shows the change in behavior for a binary choice under different values of 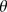 and 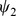 (for clarity, Figure 3 shows just the proportion of one of the two choices). In varying these two parameters, we find variation not only in final outcomes after 30 time steps, but in the dynamics of choice as well (Figure 3). When 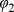 is negative, for example, we move toward social independence, and, for positive 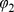, decisions tend to be made socially. Similarly, when 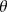 is negative, we move south toward ambivalence, and, as 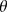 increases, we move north toward a transparency between the binary options. If 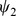 is low and 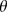 high, a member (or a whole group) might be able to choose something different from the norm. This event, however, becomes rarer as social influence increases.

**Figure 4:**
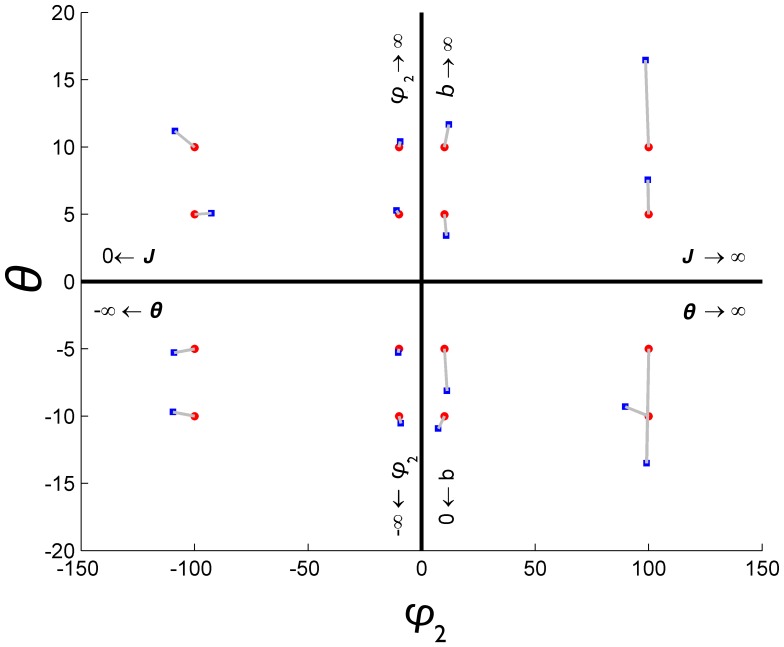
Map of the chosen values of 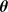 **and** 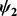 used in **Figure 3**. The red dots represent the true parameter values and are linked to their corresponding nonlinear least squares estimates in blue.

We then used the modelling to explore how the parameters 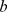 and 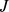 can be estimated from the simulated data. Figure 4 shows how some estimates of the parameters, based on the data generated via simulation, vary as we move along the axis on the map displayed in Figure 1. We use a nonlinear least squares (NLLS) method to estimate 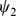 and 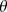; for 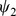 large (Figure 3, bottom row), for example, estimating 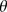 accurately is nearly impossible because there is little variation in behaviour as the social dominance of the group dominates individual utilities.

## Discussion

In these tests, we found that estimation is reasonable for 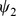 but less precise for 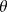. We see the source of this “weak identification” problem in equation 5 where, because 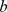 multiplies 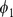 and 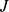, there can be difficulty in disentangling parameters in 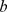 from 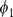 and parameters in 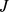 unless we have the right kind of specifications of 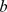 and 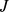 as well as variation in the observables that go into estimating their parameters. In equation 11 we can also see the challenge in disentangling the size of 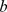 for the size of the variance of the random variable on the right-hand side (multiplying numerator and denominator by a scalar cancels out). This suggests that the variance of the numerator has to be normalized to one, say, in order to identify parameter theta in 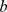.

We note that the inability to correctly estimate 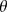 for large values of 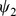 is not important because the uncertainty around 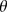 when 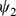 is large shows that payoff/costs are irrelevant when social influence is extremely high. In future work we will focus on how to better estimate 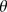. Moreover, as we focus on 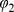 and 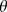, we may consider that 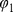 is unnecessary. We prefer to keep 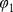 embedded in the model, as it allows for *a priori* assumptions regarding the strength of individual vs social learning — from 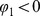 (aversion to choice 1) to 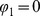 (no individual bias) to 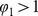 (bias towards choice 1). Further, removing 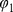 would change the inference on 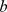 and 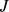; that is, 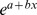 and 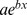 can be equivalent representations when 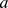 and 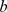 are positive, but otherwise the relevance of 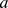 depends on the magnitude of 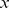 in the former and allows negative outputs in the latter.

The simple bi-axial map of behavior in Figure 1 aims to extract from aggregated data the transparency of decisions (north–south) and the extent to which a behavior is acquired socially versus individually (east–west). We have proposed a means by which to parameterize the functions that underlie the map and thus estimate paths through it. Rather than assume how well agents are informed in their learning, we can let transparency of choice be a variable parameter in our models, with the aim of using the models to infer transparency of choice from real data [19], [23]. For example, we might have a vector 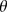 such that the transparency of choice would be parameterized as the specification 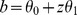.

A hypothetical real-world example for 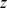 might be the fraction of unvaccinated in group 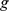 at time 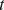. We would consider an idealized binary choice of whether or not to get vaccinated at time 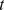, where 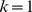 would designate vaccinated and 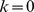 designate not vaccinated. In this example, 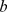 grows linearly with 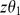, which would imply that the more unvaccinated there were in the group, the more transparent the decisions would become about vaccination. Other binary-choice examples might include whether or not to use contraception, smoke, use hand sanitizer, or perhaps the fertility decision of whether or not to have a child.

Social-influence studies that treat transparency of choice as a variable suggest that it has a complex interaction with social learning. Some of this interaction might be captured, for example, by a specification such as 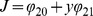, by which, assuming 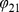 is positive, the presence of social influence increases with the scalar 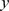. Hypothetical real-world examples for a group-specific 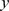 include the fraction in the group that are high income or perhaps the Gini coefficient of the group. Given the formulation for 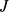, the scalar factor 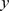 then affects the social influence associated with other observables.

These parameters relate to debates on modelling fertility decisions, for example, as explanations range from an intrinsic individual utility decision [27] versus the social influence of the frequency of a particular fertility level in their local community [32]. For example, it may be that poor, uneducated women living in a wealthy, educated group tend to adopt the low-fertility level of the group rather than the higher fertility that would otherwise be associated with their low income and low education as individuals [32]. In this case the social-choice transparency, 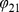, might reflect the tendency to have the same number of children as other mothers, whose success and/or education has become more socially visible.

Fertility research also generates the sort of long-term, time-stratified demographic datasets that are appropriate to our proposed estimation method for the map. More generally, the growth of so-called “big data” on collective decisions also seems suited to this map [19], which links different scales of analysis, such as the microprocesses that produce observed scaling relationships in social-network formation [33].

As new digital technologies filter and search social influences and information, transparency of choice may be increased, but conversely if agents are overwhelmed the online deluge of information, options, and social influences [34], [35], then transparency of choice, 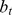, may decrease (by decreasing 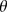 and/or 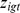). This may be central to herding effects in online product ratings [36], for example. Also, the transparency of payoffs may well be changing for many health decisions - the rapidly changing conditions of the modern world may effectively lower 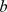 as the connection between the decision and its actual future payoffs are obscured by the “noise” of socio-economic change. Seemingly straightforward social interventions may therefore have unanticipated consequences [37].

The dimensions of the map are also relevant to studies that compare technological complexity with population size [38]–[41], which assumes relatively transparent individual and social learning. Adding agents who are uninformed (payoffs not transparent) tends to cause a group consensus to regress to a single mode [24], [42]. When it is much easier, and less costly (essentially free), to see what others do, then the balance could shift to the east and south. When survey respondents, for example, can see the aggregated guesses from other people, they simply change from their original, individual guesses in linear proportion to the distance from the group mean [43].

## Conclusion

Having presented a two dimensional map (Figure 1) as a schematic abstraction of human decision-making [19], we have now gone further toward making this into an empirical tool to project population-scale decision data onto axes of social influence and transparency of choice. As the decision maker proceeds from south to north, the precision of understanding which choice is best increases. As the decision maker moves from west to east, the strength increases of social influence or peer group influence on which choice is best. Starting with a basis in discrete-choice modeling with social influence, we have discussed how a path through the map for a group of decision makers can be estimated from data sets. Through experiments with artificial data sets, we showed how the suggested estimation methods work and how parametric specifications can be estimated. For smaller datasets, we recommend maximum likelihood as the best way to estimate a path through the map, and then for larger datasets it becomes possible to use NLLS as the estimation method.

The map can now be applied to real-world case studies, especially those that feature large, time-stratified demographic data sets on binary decisions, such as those regarding health decisions. The parametrization we have presented allows us to extract, from these sorts of datasets, locations on the map representing degree of social learning and transparency of choice. As we apply this method in the future, we may be surprised to find that standard, universal assumptions regarding certain decisions may be becoming less appropriate, as the nature of such decisions changes through time or in different cultural contexts.

## Methods

### Artificial data generation

We generated data for 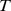 periods, 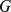 groups with 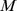 members each as follows. We first generated a random noise component, 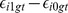, for each agent choice over the time span, which means 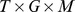 logistic random variates with mean 0 and variance 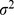. We then simulated variability in payoffs and social influence for all of these choices as well. In doing so, we generated three sets of 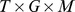 normally distributed random numbers, each with time varying means and variance, one set for the 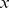
's, one set for the 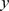
's and one set for the 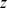
's. In this case we allowed the means of 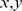, and 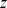 to increase over time, by choosing 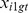 and 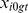 from normal distributions with mean 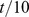 and variance 0.1, and choosing both 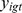 and 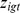 from normal distributions with mean 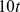 and variance 1. In generating these artificial data sets, we found that our simulation outcomes were well determined after 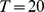 time steps, during which the effect of varying group size and members per group was minimal when both 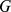 and 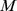 are greater than 100. We then specified the social-influence and transparency of choice functions as indicated in equation 7.

### Functional forms and estimation

In order to identify parameters of the model that describe movements across the map, we need to separate transparency of choice from social influence. To do this, we start with the north–south axis of the map (the transparency of choice) and then add, via the east–west axis, social influence on individual choices. We can start with the north–south axis. In discrete-choice theory, we assume we have a certain number of choices available and a certain amount of utility that is divided up among those choices. We then effectively toss the choices randomly into bins of a certain utility and find how many choices we expect in each bin. To start, consider a population of individuals making a binary choice (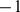 or 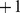), each seeking to maximize payoff function 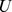:
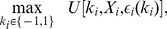
(8)in which 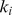 represents the binary choice and 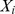 represents covariates of agent 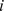 such as family, peer group, previous choices, or education level. The parameter 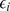 represents idiosyncrasies, which are treated as random, even if privately sensible to each individual agent. Following [31], we will assume that the values of 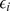 are what are known as Independent and Identically Distributed Extreme Values (IIDEV).

**Figure 5:**
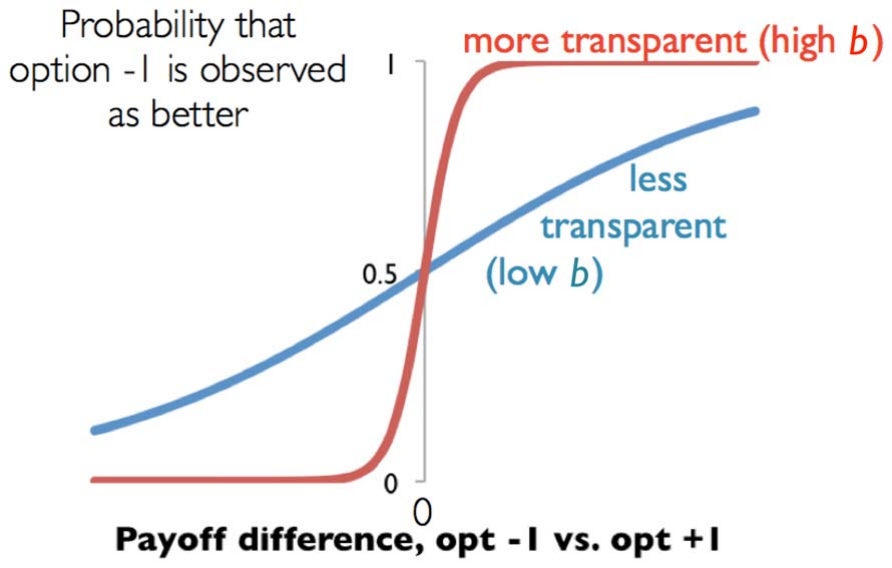
From **equation 9**, showing how the probability that option 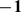 (versus option 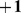) is chosen depends on this payoff difference 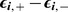, two values of 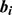.

For a given individual, a standard approach assumes the probability to make a particular choice is equivalent to the probability that the difference in idiosyncrasies, 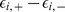, is less than some threshold 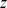:
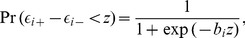
(9)where 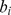 is transparency of choice for agent 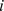. To illustrate, Figure 5 shows, for two values of 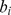, how the probability that option 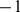 (versus option 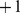) is chosen depends on this payoff difference 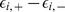. The probability transition is more abrupt or decisive for the higher value of 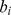 (farther north on our map), representing greater transparency of choice.

Our use of the Fermi/Boltzmann function as our equation (9) is fairly standard in discrete-choice theory, but it is also seen in some studies of evolutionary games in finite populations, in which a “temperature,” or “noise,” parameter is varied (e.g., taken to zero) in order to characterize the equilibrium in terms of cooperators in the population. The same function has been used, for example, to model the probability of outcome between two randomly selected individuals playing Prisoners Dilemma or related pairwise game [44], [45]. In that case, the parameter (analogous to temperature in the Boltzmann function) is intensity of selection rather than our transparency of choice, which operates on the payoff difference. The difference in our approach from game-theoretic approaches is twofold. First, rather than play pairwise games, agents choose among available options and the model relates how well choice popularity corresponds to covariates among the individuals of the population. Second, our focus is on econometric identification of parameters and estimation of parameters as well as on the ability to retrieve the model parameters from noisy data. In particular, we are interested in estimating the intensity of selection as a function of observable covariates. This effort appears new to the literature on estimation of social influences on choice, and it raises difficult identification issues that we have addressed through simulation methods. We developed this approach in order to show that our method works before applying it to field data.

Although this established approach does not model social influence directly, it has been used as a baseline to infer it from appropriate datasets. Aral et al. [46], for example, applied this to daily data on the social network links and the date when individuals downloaded a certain mobile-service application (app). Aral et al. [46] considered individuals of similar propensity, 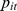, to have adopted the app by time 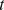, which for agent 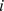 was estimated using a logistic regression equivalent to: 
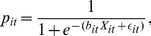
(10)where 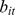 functions as transparency of choice and 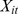 is a vector of observable characteristics and behaviors for agent 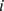 at time 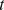 (we have subsumed one of their other parameters into the idiosyncrasies term 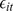). Having collected data over a 4-month period, Aral et al. [46] were able to distinguish homophily — the tendency of similar individuals to associate with each other — from genuine influence (roughly 50/50 in their final estimation).

Now, to build from this background to an explicit consideration of social influence and transparency of choice, suppose that we have 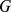 different subpopulations, each with 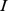 individuals. Within each population 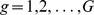 individuals are considered potential peers. Suppose we have observed covariates for all dates 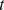 and each agent 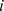 in every group 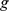, as well as the estimated propensity to be vaccinated, denoted by 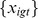. Also suppose, based on previous studies, we have another set of social-influence covariates, 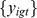, on agent 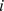 and group 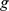 at date 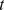, and yet another set of covariates, 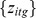, that relate how variable the choices were through time.

Our goal is to plot 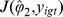 and 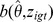 for each agent 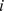 for each date 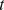 for each group 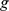 on the map, by which we could describe a temporal path for each agent 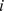 in each group 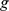. We start with the scalar covariate case where choice number 1 is made over choice 0 at date 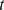. This happens when the utility of choice 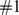 versus choice 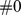, comprising the individual and social-choice components, exceeds the random variable given the choice transparency, i.e.,
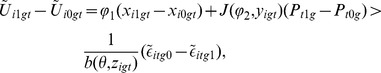
(11)where, again, the covariates, 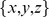, are all positive, one-dimensional scalars. Note that 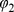 and 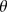 can be either positive or negative. Table 1 (below) summarizes the different parameters and variables involved. The parameter vector, 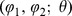, represents the intrinsic and social sensitivities, given the transparency of payoffs. The estimates of these from the data set are denoted 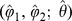
— we use “hats” to denote estimates.

With sufficient data on 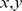, and 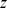 from a particular case study, we can estimate the parameter vector, 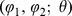, of the structural model in equations 3 and 5 using the observed fractions 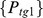 of vaccinated individuals in group 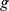 at date 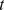 (recall that the estimates are denoted by 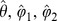). The model predicts that agent 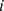 in group 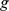 gets vaccinated at date 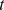 if the difference in noisy payoff 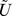 is greater than zero. In other words, the probability of positive payoff for vaccination, 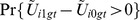, is equivalent to the probability favoring the intrinsic plus social payoffs of parenting over the random idiosyncrasies of choice:
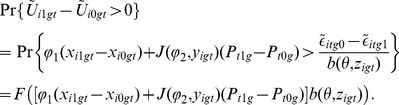
(12)


Here, 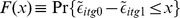 is the cumulative distribution function of the random variable, 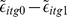.

### Functional forms for 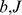


To specify 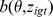 we might start with the simple specification 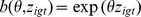, with 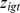 representing the variability of choices through time 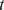. With this specification, the larger 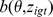 is, the less variable the choices of agent 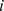 are through time.

We can then discuss several different specifications of the social-influence function 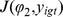. To work within the borders of the map we might, for example, specify the social-influence function as
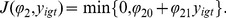
(13)


This function allows 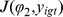 to take the value zero with positive probability, and we require the function 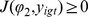, i.e., to not allow 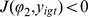, so that the farthest west part of the map corresponds to 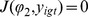. In the absence of social influence, the value of 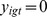. If we assume that 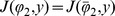 for an open set of 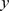
's implies 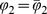, and that 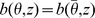 for an open set of 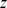
's implies 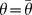, then the absence of social influence 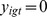 implies
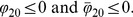
(14)


Further, we see that if the data have a wide enough range over individuals, groups, and dates, of values of 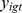, it must be the case that
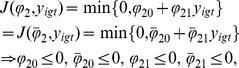
(15)i.e., social influence is zero for all values of the parameters that cannot be specified by the data alone. This level of identification can be enough when we simply want to determine the strength of social influence over time for different individuals and groups in different choice settings. We discuss another specification of the social-influence function 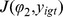 in Section 5, where we test the estimation procedure on artificial data.

### Estimation methods

In order to consider two popular estimation methods, Maximum Likelihood (ML) and Non-Linear Least Squares (NLLS), we consider a binary decision via equation 12, with the probability statement
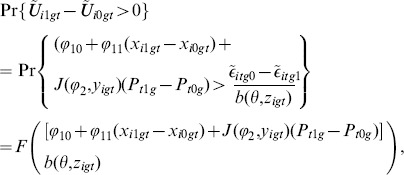
(16)where 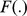 is the cumulative distribution function of the random variable, 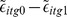. We have added a constant term, 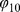, and a slope term, 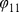, in equation 16 to capture variation among agents in how they respond to different alternatives, independent of social influences.

Now, denote by 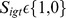 the random variable, which is either 1 if agent 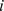 in group 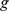 succeeds (or chooses “yes”) at date 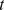 or 0 if agent 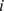 in group 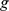 fails (or chooses “no”) at date 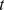. From equation 16 we can write the likelihood function for the probability of 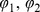, and 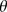, given the real-world data ([47], section 17.3), which relates to how well the model predicts all the observed successes (yeses) and failures (nos):
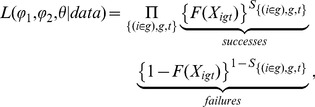
(17)where 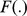 is the cumulative distribution function of the random variable, 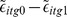, and
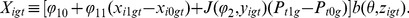
(18)


In the standard versions of estimation of discrete-choice models, the transparency of choice, 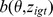, is typically assumed to be constant (absorbed into the other parameters by a normalization convention). We are interested in how 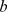 varies, however, so we must modify the conventional textbook approach [47]. One popular way of proceeding is to formulate dynamic discrete-choice models [48], which often use Markov chain formulations or hazard-function formulations. However, for simplicity we wish to remain as close as possible to the static framework with independent stochastic drivers. Therefore, we shall work with the likelihood function (equation 3.7) above, where the ultimate stochastic drivers are IIDEV across individuals, groups, and dates.

In the scalar case, formulas for the partial derivatives of the likelihood function with respect to 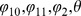 are straightforward. The maximum-likelihood estimator, at the peak of the likelihood function, is found by setting these four partial derivatives of the likelihood function equal to zero. This yields four nonlinear equations in four unknowns. When one takes these four partial derivatives and sets the four resulting equations equal to zero, one will see that when, for some reason, the social-influence function is always zero, then the pair 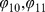 is determined only up to scale. The nonlinearity of the social influence helps resolve this particular identification problem.

If the social-influence function is zero, however, or restricted to be zero, we normalize the four equations by dividing the equations by 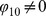, which can be further simplified by setting 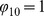 and solving the remaining three equations for 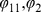, and 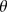. Three nonlinear equations in three unknowns is still more challenging than simple Ordinary Least Squares regression analysis.

Packages such as Matlab or R are good for general ML estimation, which works well when we have few observations per cell and enough observations per cell to allow for logistic regression [49]. Also, ML estimation does not assume that errors follow a specific distribution, whereas NLLS assumes normality of the errors, and one can use least squares estimators as starting points on the ML solver. ML estimation is less demanding of data sets than NLLS [47, chapter 14], but in cases where there is a large amount of data, we can also consider NLLS estimation methods that require larger data sets. Equation 5 suggests the NLLS regression equation of the log odds of agent 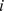 in group 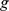 of choosing choice 1 rather than choice zero at date 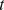,
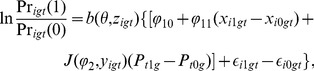
(19)in which the right-hand side again consists of transparency of choice multiplied by individual, social, and noise components (note that the noise terms 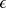 in equation 19 are part of the standard regression equation framework and are not the same as the 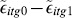 terms in the logit equations above). Here, we assume the standard regression orthogonality condition on the regression errors,
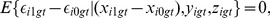
(20)so that parameter estimates are consistently estimated as sample size tends to infinity.

Note that equation 20 implies
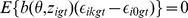
(21)by taking iterated expectations. Equation 21 assures us that if we estimate the parameters in equation 19 by NLLS, the estimates will have good properties, provided that the parameter vector, 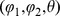, in the structural model in equation 19 is identified ([47], chapter 7). Of course, since the function induces heteroskedasticity in the residuals, 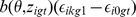, of the NLLS regression equation 19, this heteroskedasticity can be exploited to produce more efficient estimates ([47], chapter 7). To avoid some problems of large sample size and data overflow, an improved NLLS estimation process might use a growing window in time, i.e., start with points corresponding to time 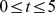 and locate a plausible region on the parameter space, add more points for time 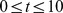 and update, and so on.

Consider the functional-form specifications for the transparency of choice function and the social-influence function,
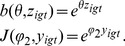
(22)


Substituting 22 into 19, NLLS proceeds by selecting the parameter vector, 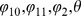, to minimize the sum of squared errors (SSE),
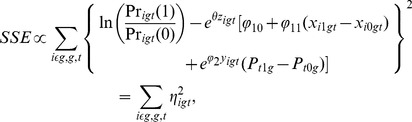
(23)where
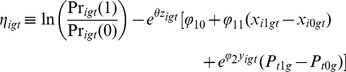
(24)


is the prediction error of the model. In other words, NLLS chooses the parameter vector to minimize the sum of prediction errors. Taking the four partial derivatives of SSE with respect to 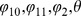, and setting all four of them equal to zero, we have the four following four nonlinear equations in four unknowns:
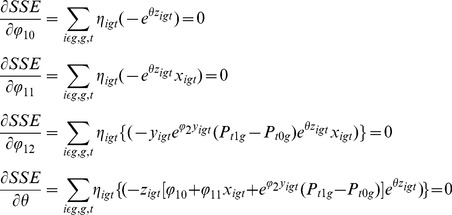
(25)


We can see that if for some reason the term 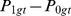 is always zero, then the pair of parameters, 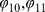, of the direct utility difference is determined only up to scale. To put it another way: if we set the social-influence function 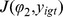 equal to zero, then the parameter pair, 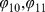, is not identified, i.e. any parameter pair 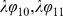 will solve the last equation of 25 with the third equation dropped for all values of 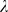. We resolve this problem if it occurs by “normalizing” by setting 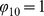 and dropping the first equation of 25. We recommend the same procedure for the ML estimation above.
